# Perceived Duration Depends Upon Target Detection in Rapid Serial Visual Presentation Sequence

**DOI:** 10.1177/2041669520981996

**Published:** 2020-12-25

**Authors:** Makoto Ichikawa, Masataka Miyoshi

**Affiliations:** Department of Psychology, 12737 Chiba University, Chiba, Japan

**Keywords:** attentional blink, temporal duration, rapid serial visual presentation, number of perceived frames, cognitive load

## Abstract

It is well known that the perceived duration for a given time period decreases with the reduction of the number of perceived events. We examined whether target detection failures in viewing Rapid Serial Visual Presentation (RSVP) sequence, caused by attentional blink, affect this reduction of perceived duration. In two experiments, trials consisted of displays of two series of RSVP sequences; in the first sequence (the comparison), two, one, or no numerals were presented as targets embedded within the string of letters, while in the second sequence (the standard), only alphabetic letters were presented. In each trial, participants judged whether the duration of the comparison is perceived as longer than that of the standard (Experiment 1), or whether the number of frames in the comparison is perceived as more than that in the standard (Experiment 2). Results showed that perceived duration was inflated with target detection, but not with the increment of presented frames although number of perceived frames was inflated with both target detection and increment of presented frames. These results suggest that perceived duration in viewing RSVP sequences is determined by the cognitive load necessary to accomplish target detection rather than by the number of perceived frames.

Previous studies have found that perception of temporal duration depends upon various factors. That is, perceived duration increased with frequency of attending to the lapse of time (Fraisse, 1984), perceived size of the stimulus (Ono & Kawahara, 2007; Thomas & Cantor, 1975, 1976), the number denoted by a stimulus (Xuan et al., 2007), the amount of energy expended by neurons required to process perceptual stimuli during the period (Eagleman, 2008; Pariyadath & Eagleman, 2007), physical load (Block et al., 2016; Lu et al., 2011) and cognitive load (Block et al., 2010) during observation, and strong emotion (Angrilli et al., 1997; Gil & Droit-Volet, 2012; Kobayashi & Ichikawa, 2016; Stetson et al., 2007; Watts & Sharrock, 1984; Yamada & Kawabe, 2011). Perceived duration of a given period varies not only with an actual lapse of time, but it is also affected by other factors, which are irrelevant to the actual lapse of time. Also, the perceived number of events during that period (Brown, 1995; Fraisse, 1984; Poynter, 1989) is a factor affecting perceived elongation of a duration. That is, if individuals experience more events during a fixed period, they tend to perceive the duration of this period as longer than the same duration which they experience filled with less events.

The present study considers the effects of the perceived numbers of events and other factors on perceived duration in observers experiencing the Rapid Serial Visual Presentation (RSVP) display in which multiple visual stimuli are presented in rapid succession. In viewing an RSVP display, which includes two targets, observers often fail to detect the second target if the lag between the first (T1) and second targets (T2) is less than 500 ms. This failure in detecting the T2 is known as attentional blink (AB; Raymond et al., 1992). Several studies account for the occurrence of AB by positing a cognitive limitation of attentional resources that is allocated to the T1 at the expense of the T2. That is, Shapiro et al. (1994) claim that a large part of attentional resources are allocated to the T1 in a sequence, and this results in a resource depletion for the following items in the RSVP stream; the results, then is an AB. More specifically, Chun and Potter (1995) claimed that an AB occurs when the T2 arrives while a limited cognitive resource is busy with the T1 so that the T2 is deprived of the same resource. These accounts assume that a capacity limitation may be involved in occurrence of the AB. However, in some models, capacity limits play no role in the generation of the AB. For instance, the temporary loss of control theory (Di Lollo et al., 2005; Kawahara et al., 2006) proposed that a distractor would disrupt an input filter, which governs the central processing of incoming stimuli by passing target and excluding nontarget items, and that this disruption by a distractor immediately after T1 may cause the AB. In the boost and bounce theory (Olivers & Meeter, 2008), working memory employs an input filter that enhances (boosts) the processing of stimuli that match the target set and inhibits (bounces) stimuli that do not. This strong but transient inhibition of subsequent stimuli would cause the AB. Also, the episodic simultaneous type/serial token model (Wyble et al., 2009, 2011) proposes that targets presented separately are stored in episodically distinct working memory representations and that the AB reflects the suppression of attention which provides this separation; an uninterrupted sequence of targets can be encoded, but following a gap in the sequence, attention is briefly switched off to divide the encoding process into two sequential episodes. This model has advantage over other models in explaining the prolonged sparing and order reversals for reported targets (e.g., Dux & Marois, 2009). 

The present study is concerned with the perceived temporal duration when observers view the RSVP sequence, especially when the AB occurs. In occurrence of the AB, observers fail to detect the T2 when viewing a RSVP display. One can assume that the number of perceived frames when T2 is missed would be fewer than the number of perceived frames without missing it. Accordingly, one may assume that the perceived duration of an RSVP with the AB would be shorter than the perceived duration without the AB, because the AB should eliminate the perception of an event. This issue was investigated by Herbst et al. (2012). They presented RSVP streams and combined a target detection task with a prospective duration judgment task. That is, on each trial, a standard sequence without targets was followed by a test sequence, which contained one or two*-*target stimuli. Participants were asked to count the targets in the test sequence and judge the duration of the test sequence relative to the standard sequence. Their results showed that the perceived duration was reduced when the AB occurs. They concluded that the number of subjectively perceived target stimuli (not the number of objectively presented targets) determines the subjective duration of the entire RSVP sequence. In addition, they claimed that the attentional selection (or spontaneous fluctuations of vigilance), which is related to the number of perceived targets, prolonged perceived duration for the RSVP sequence.

In this previous study (Herbst et al., 2012), the difference between the trials in which the AB occurs and trials in which it does not is not limited to the number of perceived frames. For instance, on two*-*target streams, the cognitive load imposed to the visual system when all targets are detected should be higher than the cognitive load imposed when detecting only one target. Cognitive load which is imposed on the visual system in processing the visual stimulus would affect temporal duration (Block et al., 2010). Therefore, in the present study, to examine how cognitive loads imposed on the visual system in accomplishment of target detection affect duration perception, we introduced a condition that presented no target (the No-target condition), along with One-target and Two-target conditions, in which RSVP sequences included one (only T1) or two targets (T1 and T2), respectively. If the cognitive load imposed in proper accomplishment of target detection determines perceived duration for the RSVP sequences, the perceived duration would be inflated by detecting all the targets in RSVP sequence, regardless of the number of detected targets. However, as mentioned preciously, Herbst et al. (2012) proposed that attentional selection, which is related to the number of perceived targets, prolonged perceived duration for the RSVP sequence. Therefore, they would predict that perceived duration for the RSVP sequence increases linearly with the number of reported targets for the No-target condition, One-target and Two-target conditions. To determine which is the case, we conducted an experiment (Experiment 1) in which we asked participants to judge whether the duration of the comparison RSVP sequence, which may include targets, is temporally longer than that of the standard RSVP sequence, which includes no target. In each trial in Experiment 1, observers reported the target(s) which they detected, and they reported which of the first and second sequences looked longer.

In addition, we examined the relationship between the perceived duration and perceived frame number for RSVP sequences. Although Herbst et al. (2012) claim that the number of subjectively perceived target stimuli determines subjective duration, they did not directly examine the relationship between the perceived duration and perceived number of frames for RSVP sequences. In Experiment 2 of the present study, to directly assess the relationship between the perceived duration and the perceived number of frames for RSVP sequences, we asked participants to judge which of a comparison RSVP sequences or a standard RSVP sequence includes more frames. If the perceived duration for the RSVP sequence is determined by the number of subjectively perceived target stimuli, as Herbst et al. (2012) claim, then both the perceived duration and the perceived number of frames in viewing RSVP sequences should vary with number of presented and detected targets and with number of presented frames in the RSVP sequence. To assess the effects of number of objectively presented frames on perceived duration and perceived frame number for RSVP sequence, we introduced the difference in the actual number of frames between the comparison and standard RSVP sequences, as well as the variation for number of objectively presented frames in those RSVP sequences.

## Experiment 1

We conducted the first experiment to investigate how the cognitive load, which is imposed on the visual process in processing targets in viewing RSVP sequence, affects the perceived duration for the RSVP sequence. In Experiment 1, on each trial, two series of RSVP sequences were presented successively; the first sequence was a comparison stimulus, which could include targets, and the second one was a standard stimulus, which included no target. In the first RSVP sequence, participants were told to detect targets, that is, numerals among the series of alphabetic letters. Next, they conducted a relative temporal duration judgment task between the first and second RSVP sequences.

### Methods

#### Observers

Eight naïve graduate or undergraduate students served as observers. All had normal or corrected-to-normal visual acuity and were right-handed.

#### Stimuli and Apparatus

A personal computer (Dell Precision T3400) presented stimuli on a CRT display (FlexScan T766, Eizo, 19inch, 85 Hz). The observer sat on a chair in front of a desk (80 cm in height), with the head fixed on a chin rest. A computer keyboard was placed by the observer’s right hand. Viewing distance was 57 cm.

**Figure 1.**
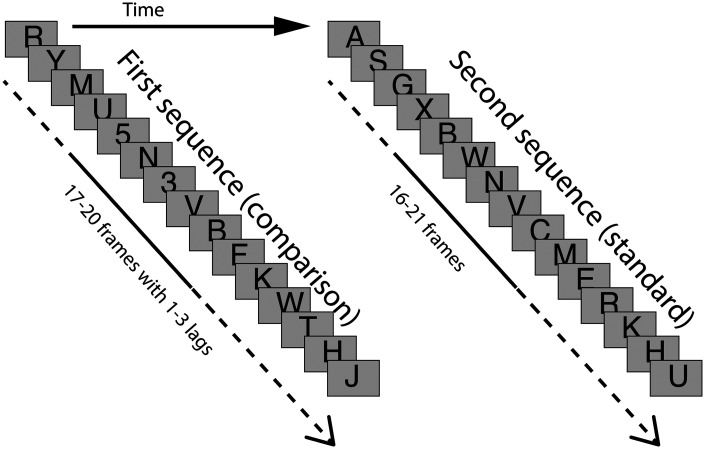
Sequence of stimulus presentation in Experiment 1.

On each trial, two RSVP sequences with black uppercase alphabetic letters (1.47 ×1.47 deg, 0.03 cd/m^2^) were presented on a gray background frame (1.0 cd/m^2^; Figure 1), while B, I, O, S, and Z were eliminated from the sequence due to resemblance to certain Arabic numerals. The first RSVP sequence was a comparison sequence, which might include black Arabic numerals (2, 3, 4, 6, 7, 8, or 9) as targets. The second sequence was a standard sequence, which presented successive frames containing only alphabetic letters. Each frame was presented for 70 ms with 23 ms of interstimulus interval. On one hand, the length of a comparison sequence ranged from 17 to 20 frames. On the other hand, the total length of the standard sequence was one of the three conditions; relative to the length of the comparison sequence, the total length of the standard sequence was either the exact length of the comparison sequence, or it was one frame shorter or one frame longer than the preceding comparison sequence.

There were three conditions for the comparison sequences. That is (a) the No-target condition in which only uppercase letters were presented (3 [actual frame difference between the comparison and standard sequences] × 16 [repeat] = 48 trials), (b) the One-target condition in which one Arabic numeral appeared as a target (T1) within series of uppercase letters (3 [actual frame difference between the comparison and standard sequences] × 16 [repeat] = 48 trials), and (c) the Two-target condition in which two Arabic numerals appeared as the first (T1) and second targets (T2) within a series of uppercase letters, and a lag between the T1 and T2 was 1, 2, or 3 frames (3 [actual frame difference between the comparison and standard sequences] × 3 [lag] × 16 [repeat] = 144 trials). In the One-target and Two-target conditions, at least four letters were presented before the first target (T1) appeared.

#### Procedure

At the beginning of each trial, a black cross (1.0 × 1.0 deg, 0.03 cd/m^2^) was presented as a fixation point at the center of the display. Observers were told to fixate on this black cross and to press the space key to start the presentation of the first RSVP sequence. Following the participant’s keypress by 500 ms, the first RSVP sequence (comparison sequence) appeared above the fixation cross. While fixating on the black cross, observers then pressed the space key again to start the second RSVP sequence (standard sequence). Then, 500 ms after the participant’s keypress, the second RSVP sequence appeared, and 500 ms after the end of the standard sequence, a frame appeared that asked the participant to press key(s) that correspond to the detected target(s) with the presented order and then press the return key. They were asked to press the “I don’t see any target” key on the response keyboard when they did not see any target(s). Then, they reported via keypress, which of the first and second sequences looked longer; if they judged the duration of the first sequence was longer than that of the second sequence, they pressed the “f” key; if they judged the duration of the first sequence was shorter, they pressed the “k” key. They were instructed to base their duration judgment on their subjective lapse of time and not to count seconds in viewing RSVP sequences. The order of conditions was randomized. In addition, the length of the first RSVP was varied within the range from 17 to 20 frames in random order.

### Results and Discussion

**Figure 2.**
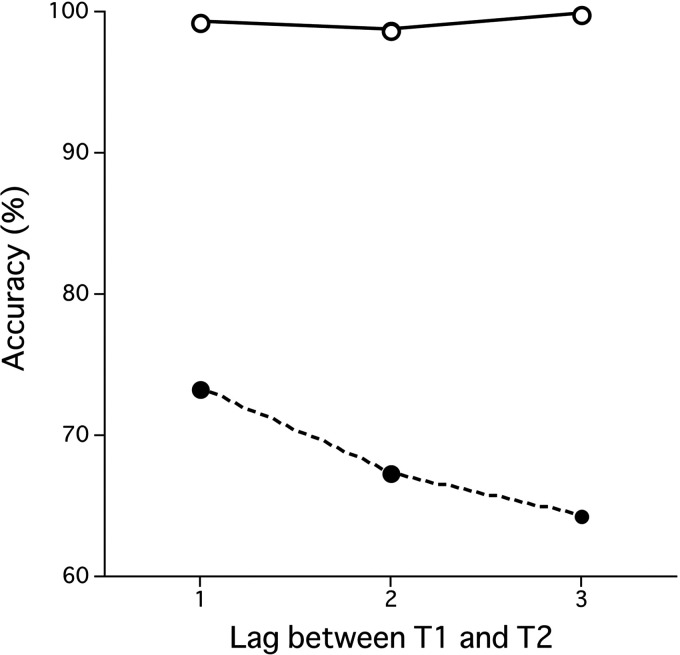
Accuracy in the target detection task for the Two-target condition in Experiment 1. The vertical axis is the frequency of correct response in the target detection task (%). Open symbols show the T1 accuracy, and closed symbols show T2/T1 accuracy. The horizontal axis is the lag between the first (T1) and second targets (T2) in the comparison RSVP sequence.

For both the Two-target condition and One-target condition, if observers reported wrong numbers as targets, or if they missed the target, we counted the report as an error in the target detection task. Also, if observers reported any numbers as targets in the No-target condition, we counted such reports as errors. The error rate in the One-target condition was 9.1% which includes not only missing T1 but also false alarms for the T2 (4.7%). The error rate in the No-target conditions was 5.2% which includes single (3.4%) and double (1.8%) false alarms. For the Two-target condition, frequency of the T1 detection error was 0.8%, while the frequency of the T2 detection error with correct T1 detection (frequency of the AB) was 31.8%. Figure 2 shows accuracy for the T1 and accuracy for T2 when T1 was correctly detected in the Two-target condition.

**Figure 3.**
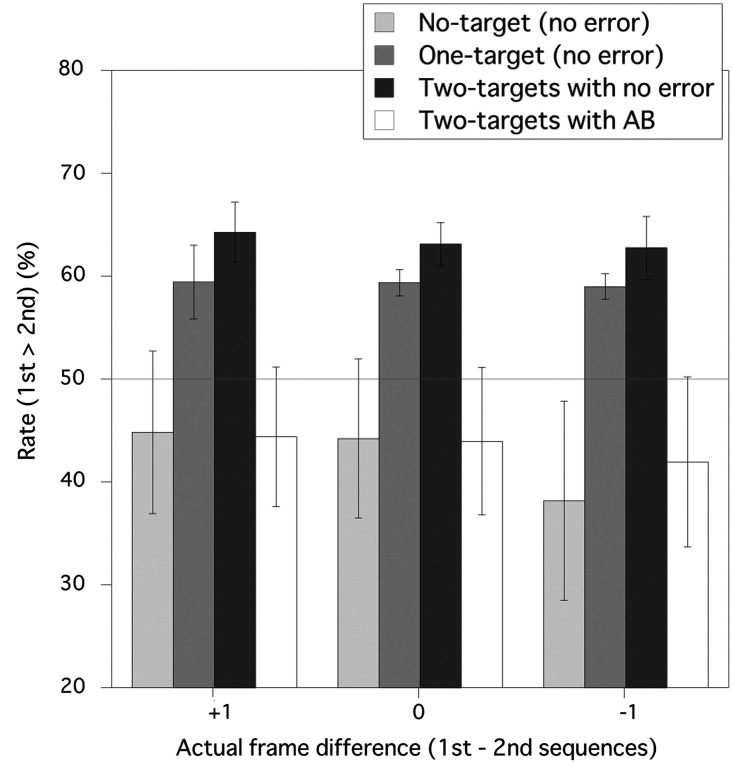
Duration judgments of the comparison sequence relative to the standard sequence in Experiment 1. The vertical axis is the frequency of the trials in which observers perceived the duration of the first RSVP sequence (comparison stimulus) as longer than the duration of the second RSVP sequence (standard stimulus). The error bars show 95% confident limits. A horizontal gray line shows the chance level (50%). AB = attentional blink.

Figure 3 shows the mean frequency of the trials in which participants perceived the first RSVP sequence as longer than the second sequence for each for the No-target condition, One-target condition, and Two-target condition. For the No-target and One-target conditions, the results were from the trials in which participants correctly responded. For the Two-target condition, the results were presented separately for the case in which both T1 and T2 were correctly detected and the case in which only T1 was correctly detected (AB occurred). Results from other types of error for the Two-target condition were not presented in Figure 3 because those errors were relatively rare. These four categories, the No-target condition, One-target condition, Two-target condition with no error, and Two-target condition with AB, are treated as the “target detection cases” in the following analysis. A two-way repeated measures analysis of variance (ANOVA) with the target detection case (4; the No-target condition, One-target condition, Two-target condition with no error, and Two-target condition with AB) and the actual frame difference between the comparison and standard sequences (3; +1, 0, and –1) as factors for the duration judgment found a significant main effect of the target detection condition, *F*(3, 21) = 7.141, *p* = .0017, while both the main effect of the actual frame difference, *F*(2, 14) = 0.495, *p* = .6200, and the interaction of these two factors, *F*(6, 42) = 0.270, *p* = .9478, were not significant. A post hoc Ryan test for the main effect of the target detection case showed that the perceived duration for both the No-target condition and the Two-target condition with AB was shorter than those for the One-target condition and Two*-*target condition with no error. There was no significant difference between the One-target condition and Two-target condition with no error. These results indicate that the perceived duration of the first sequence increased in terms of target detection although there was no significant effect of the actual frame difference. In the No-target condition, One-target condition, and Two-target condition with no error, no target was missed, and therefore, the number of perceived frames should be the same. However, target detection inflated the temporal duration for the RSVP sequences regardless of detected target number. Also, although the perceived frame number for the Two-target condition with AB should be less than that for the No-target condition with no error, perceived temporal duration for the former one was almost the same as that for the later. These results were not compatible with the claim by Herbst et al. (2012), namely that the number of subjectively perceived target stimuli determines subjective duration in viewing RSVP sequence.

Means and 95% confidence limits in Figure 3 show that the rate of trials in which the comparison sequence was perceived as longer than standard sequence was significantly higher than 50% if all of the targets in the sequence were correctly detected regardless of number of detected targets. These results indicate that the temporal duration would be overestimated if all of the targets in the sequence were correctly detected. However, that rate could be lower than 50% for the No-target condition if the number of presented frames in the comparison sequence was fewer than that in the standard sequence. In other cases, including the Two-target condition with AB, that rate was not significantly less than 50% level. This result implies that the perceived duration was hardly underestimated when T2 was missed (i.e., AB occurred).

We found that the perceived durations for the One-target condition and Two-target condition with no error were both longer than that for the No-target condition although number of perceived frames was the same among these cases. Importantly, there was no difference between the One-target and Two-target conditions with no error in the post hoc tests after ANOVA. To confirm the absence of difference between the One-target condition and Two-target condition with no error, we calculated the Bayes factors (Lee & Wagenmakers, 2014) under the hypothesis that the ratio of the Two-target condition with no error is larger than that of the One-target condition (BF_−0_) for each of +1, 0, and –1 conditions; 0.171, 0.139, and 0.173, respectively, by the use of Bayesian *t* test in terms of JASP (JASP Team, 2020). These values of the Bayes factors are within the level of the moderate evidence for H_0_ (1/3; Keysers et al., 2020); the ratio of the Two-target condition with no error is not larger than that of the One-target condition with no error.

In the Two-target condition with AB, although T2 was missed, T1 was successfully detected, identified, and registered. However, the perceived duration for this case was shorter than that for the One-target condition. This result suggests that the quite large difference in the perceived duration between these two cases is a consequence of the failure in processing the whole targets in a RSVP sequence. This problem will be discussed together with the results of Experiment 2.

## Experiment 2

In the first experiment, we found that the perceived durations for the One-target condition and Two-target condition with no error were longer than that for the No-target condition although number of perceived frames should be the same among these three cases. In addition, although the perceived frame number for the Two-target condition with AB should be perceived as less than that for the No-target condition, the perceived temporal duration for the former was almost the same as that for the later. These results are inconsistent with the claim by a previous study (Herbst et al., 2012) that simply the total number of subjectively perceived target stimuli determines an observer’s subjective duration in viewing RSVP sequences.

However, in the first experiment, as well as in Herbst et al. (2012), the relationship between the target detection and number of perceived frames was not examined. It remains possible that the relationship between the target detection and perceived duration, which was observed in the first experiment, is determined by the relationship between the target detection and number of perceived frames. To examine this possibility, participants conducted a target detection task for the first RSVP sequences, which resembled the design of Experiment 1. However, after this in Experiment 2, participants then conducted a relative frame number judgment task in which they compared the total frame numbers of the first and second RSVP sequences. If we find the results similar to those found in the first experiment, then we may conclude that the number of subjectively perceived target stimuli determines subjective duration in viewing RSVP sequences.

### Methods

#### Observers

Ten naïve graduate or undergraduate students took part in Experiment 2. Five of them participated in Experiment 1.

#### Stimuli and Apparatus

A personal computer (HP ProDesk 600 G2 SF) presented stimuli on a CRT display (MITSUBISHI Diamondcrysta RDT196LM, 19 inch, 60 Hz). The observer sat on a chair in front of a desk, with the head fixed on a chin rest. A computer keyboard was placed by the observer’s right hand. Viewing distance was 57 cm.

We used the stimuli similar to those of Experiment 1. In each trial, two RSVP sequences with black uppercase letters (1.47 × 1.47 deg, 0.005 cd/m^2^) were presented on a gray background (1.885 cd/m^2^). B, I, O, S, and Z were excluded from the sequence due to resemblance to certain Arabic numerals. The first RSVP sequence was a comparison stimulus, which might include black Arabic numerals (2, 3, 4, 6, 7, 8, or 9) as targets. The second RSVP sequence was a standard stimulus, which presented only alphabetic letters. Each frame was presented for 83 ms with 17 ms of interstimulus interval. On one hand, the length of comparison RSVP sequence ranged from 17 to 20 frames. On the other hand, the total length of standard RSVP sequence was one of the three conditions; relative to the length of the comparison sequence, the total length of the standard sequence was either the exact length of the comparison sequence, or it was one frame shorter or one frame longer than the preceding comparison sequence.

There were three conditions for the first RSVP sequences; they were identical to the conditions used in Experiment 1: (a) the No-target condition in which only uppercase letters were presented (3 [actual frame difference between the comparison and standard sequences] × 16 [repeat] = 48 trials), (b) the One-target condition in which one Arabic numeral appeared as a target (T1) within series of uppercase letters (3 [actual frame difference between the comparison and standard sequences] × 16 [repeat] = 48 trials), and (c) the Two-target condition in which two Arabic numerals appeared as targets (T1 and T2) within a series of uppercase letters, and a lag between the T1 and T2 was 1, 2, or 3 frames (3 [actual frame difference between the comparison and standard sequences] × 3 [lag] × 16 [repeat] = 144 trials).

#### Procedures

Procedures of Experiment 2 were similar to those of Experiment 1. The only exception was that, in Experiment 2, participants conducted a relative frame number judgment task between the first and second RSVP sequences, instead of relative temporal duration judgment between them, after the target detection task for the first RSVP sequence.

### Results and Discussion

**Figure 4.**
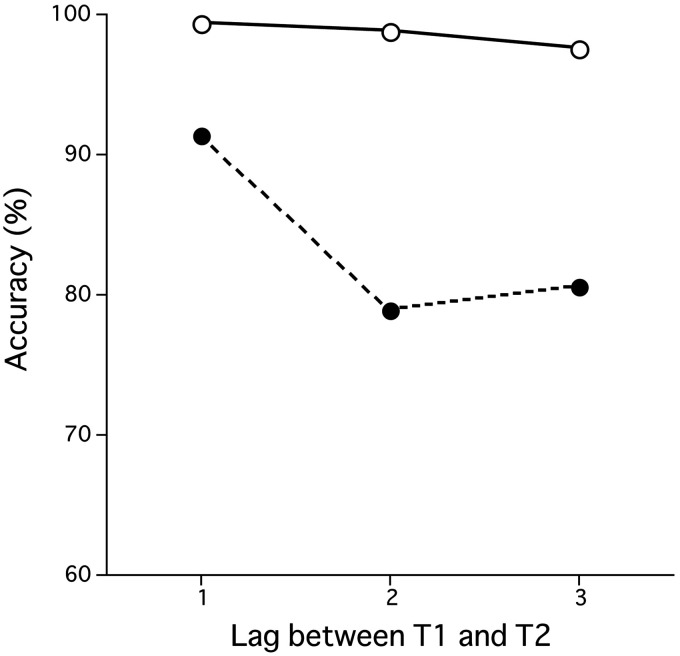
Accuracy in the target detection task for the Two-target condition in Experiment 2. The vertical axis is the frequency of correct response in the target detection task (%). Open symbols show the T1 accuracy, and closed symbols show T2/T1 accuracy. The horizontal axis is the lag between the first (T1) and second targets (T2) in the comparison RSVP sequence.

We defined errors in the target detection task for each of the Two-target condition, One-target condition, and No-target condition as in Experiment 1. The error rate in the One-target condition was 23.7% which includes not only missing T1 but also false alarms for the T2 (16.7%), which was quite high compared with frequency of the same type of error in Experiment 1. The error rate in the No-target conditions was 6.8% which includes single (4.7%) and double (2.1%) false alarms. For the Two-target condition, frequency of the T1 detection error was 1.5%, while the frequency of the T2 detection error with correct T1 detection (frequency of the AB) was 16.3%, which was relatively low compared with frequency of the same type of error in Experiment 1. Figure 4 shows accuracy for the T1 and accuracy for the T2 when T1 was correctly detected for the Two-target condition. Two of the observers rarely had AB in viewing the Two-target condition (0.23% and 0.69%), and therefore, they had no relative frame number judgment data with some actual frame difference conditions. In the following analyses, we used the data of the other eight observers.

**Figure 5.**
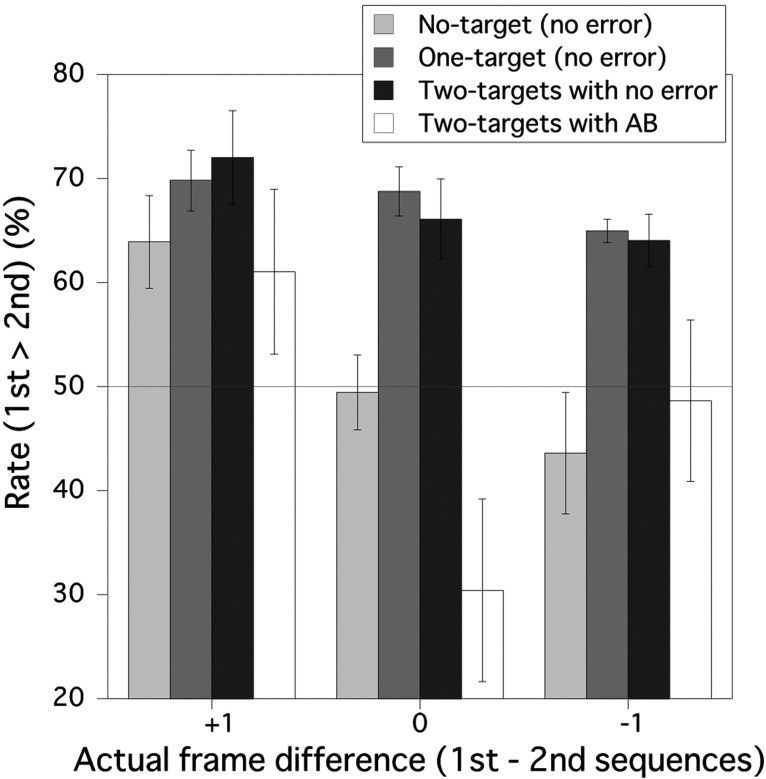
Frame number judgments of the comparison sequence relative to the standard sequence in Experiment 2. The vertical axis is the frequency of the trials in which observers perceived the number of frames included in the first RSVP sequence (comparison stimulus) as more than the number of frames included in the second RSVP sequence (standard stimulus). The error bars show 95% confident limits. A horizontal gray line shows the chance level (50%). AB = attentional blink.

Figure 5 shows the mean frequency of the trials in which participants perceived that the frame number included in the first RSVP sequence (comparison sequence) is more than the frame number included in the second RSVP sequence (standard sequence) in each of four “target detection cases”: the No-target condition, One-target condition, Two-target condition with no error, and Two-target condition with AB. Figure 5 shows the effects of the actual frame difference conditions for the No-target condition, which was not found in Experiment 1. That is, for the No-target condition (white bar in Figure 5), the frequency of trials in which the first sequence (comparison sequence) was perceived as including more frames than the second sequence (standard sequence) significantly exceeded 50% for the +1 condition, was at the same level with 50% for the 0 difference condition and was significantly lower than 50% for the –1 condition. In addition, perceived frame numbers for the comparison sequence in the +1 condition were correctly perceived as longer than the standard sequence (significantly exceeded 50%) regardless of the four target detection cases. These results indicate that the number of perceived frames increased with the increase of number of actually presented frames when no target was presented in a RSVP sequence.

A two-way repeated measures ANOVA with the target detection case (4; the No-target condition, One-target condition, Two-target condition with no error, and Two-target condition with AB) and the actual frame difference between the comparison and standard sequences (3; +1, 0, and –1) as factors for the perceived frame number found significant effects of the actual frame difference on number of perceived frames. That is, we found significant main effects not only for the target detection case, *F*(3, 21) = 10.657, *p* = .0002, but also for the actual frame difference, *F*(2, 14) = 4.982, *p* = .0232, as well as significant interaction of the two factors, *F*(6, 42) = 2.531, *p* = .0350.

For the main effect of the target detection case, post hoc Ryan tests for the main effect of the target detection case showed that the perceived duration for the No-target condition and Two-target condition with AB was less than those for the One-target condition and Two*-*target condition with no error. There was no significant difference between the One-target and Two-target conditions with no error. Results of the Two-target condition with AB confirmed that, when the visual system fails to process T2, not only temporal duration (examined in Experiment 1) but also the number of presented frames is underestimated. In addition, because no target was missed in the No-target condition, One-target condition, and Two-target condition with no error, perceived frame number should be the same among these three target detection cases. However, the number of perceived frames for the No-target condition was less than those for the One-target and Two-target conditions with no error. These results indicate that perceived number of frames, as well as perceived temporal duration (examined in Experiment 1), varies with the required cognitive load necessary to process targets in viewing RSVP sequences. The effects of cognitive load to process targets in viewing RSVP sequences on temporal duration and perceived number of frames will be discussed in the General Discussion section.

Post hoc Ryan tests for the main effect of the actual frame difference showed that the number of perceived frames for the +1 condition was significantly more than those for the other two conditions (*p* < .05). These results from ANOVA and post hoc tests, together with the results that show that the number of perceived frames increased with the increase of number of actually presented frames when no target was presented (Figure 5), imply that the perceived number of frames in RSVP sequence would vary with the number of presented and correctly processed frames.

For the interaction of the two factors, post hoc tests showed that the effects of target detection case depend upon the actual frame difference condition. That is, there was no significant difference among the four target detection cases in the +1 condition (*p* > .10). In the 0 difference condition, however, the number of perceived frames for the No-target condition was significantly less than those for the One-target and Two-target conditions with no error and that for the Two-target condition with AB was much less than the other three cases. Such a large shrinkage for the Two-target condition with AB was not observed for the perceived temporal duration in Experiment 1. In the –1 condition, the number of perceived frames for the No-target condition was significantly less than those for the One-target and Two-target conditions with no error (*p* < .05). In addition, in the –1 condition, although the actually presented frame number in the comparison sequence was less than that in the standard sequence by one frame, the perceived frame numbers for the comparison sequences in the One-target and Two-target conditions with no error were more than that for the standard sequences, and even if T2 was missed, perceived frame number for the comparison sequence was at the same level with the number of frames in the standard sequence. The Bayes factor under the hypothesis that the ratio of the Two-target condition with no error is higher than that of the One-target condition (BF_−0_) for the +1 condition was 0.231. Because the mean frequencies of the One-target condition with no error were higher than those of the Two-target condition with no error for the 0 and –1 conditions (Figure 5), we obtained the Bayes factors under the hypothesis that the ratio of the Two-target condition with no error is lower than that of the One-target condition (BF_+0_) for these conditions: 0.199 and 0.259, respectively. These values of the Bayes factors below 1/3 provide moderate evidences for H_0_; the higher ratio does not lead to a substantial inflation of perceived frame number.

The results of Experiment 2 demonstrated that the number of perceived frames varied with the number of actually presented frames, especially when no target was presented, as shown by the results of the No-target condition. However, when one or two targets were successfully detected, the number of perceived frames was overestimated, and the extent of such an overestimation might surpass the deficit in number of the actually presented frames, as shown by the –1 condition. In addition, we found that the number of perceived frames might deflate with missing of T2 in the RSVP sequence. Extent of such a deflation would be quite large if there was no difference in the number of actually presented frames between the comparison and standard sequences. These results indicate that the effects of factors which are related to target detection in the comparison sequence on the number of perceived frames would be larger than those of number of actually presented frames. The effects of factors that are related to target detection on the number of perceived frames will be discussed together with the effects of those factors on the perceived temporal duration in General Discussion section.

## General Discussion

We investigated the effects of the actual frame differences between comparison and standard sequences and target detection on perceived temporal duration in Experiment 1. In this experiment, observers reported perception of temporal duration of pairs of RSVP sequences that varied in frame factors. In Experiment 2, we investigated the effects of these factors on perceived frame number in viewing RSVP sequences. We found certain common effects of these factors on the observers’ perceived duration and number of perceived frames, as well as certain differences between them. The main common ground in the effects for the perceived duration and number of perceived frames was the effects of target detection cases. The main difference between them was the effects of the actual frame differences between the comparison and standard sequences. Specifically, both perceived duration and perceived frame number varied in a similar way with the target detection cases, but only the perception of frame numbers varied with the actual frame difference between the comparison and standard sequences. Based upon these common grounds and differences, we will discuss the basis of perception of temporal duration and perception of frame numbers in viewing RSVP sequences and also relationship between them.

We found similar effects of target detection cases on both the perception of temporal duration and perception of frame numbers. The main effect of the target detection case in ANOVA and post hoc tests showed significant differences between the results of the No-target condition and Two-target condition with AB and results of the One-target condition and Two-target condition with no error. Although the perceived frames should be at the same level among the No-target condition, One-target condition, and Two-target condition with no error, we found a significant difference between the No-target condition and those of the other two conditions. We have to point out that, compared with the variance among these three conditions, the differences between the One-target condition and Two-target condition with no error, which was reported for the perceived duration in Herbst et al. (2012), were relatively small. These results suggest that both the perceived duration and perceived frame numbers vary not only with the number of actually presented frames but also with the cognitive load required to process the target. The result of post hoc tests after ANOVA, as well as the Bayes factor analyses, that there was no difference between the One-target condition and the Two-target condition with no error implies that not simply the number of detected targets or target search itself, but maybe cognitive load, which is imposed on the visual system to detect, identify, and register the whole targets in the RSVP sequence would dilate both the perceived duration and perceived frame numbers in viewing RSVP sequence. The basis of the effects of cognitive load, which is imposed to the visual system, on the perceived duration will be discussed later.

In addition, in the Two-target condition with AB in which T1 was correctly detected while T2 was missed, both perceived duration and perceived frame number were at the same level as those in the No-target condition (with the –1 and +1 conditions), or less than them (with the 0 difference condition). Moreover, if the targets were correctly detected, regardless of the number of detected targets, the temporal duration and number of frames were inflated. Herbst et al. (2012, p. 883) claimed that “the number of subjectively perceived target stimuli (and not the number of objectively presented targets) determine subjective duration of the entire RSVP sequence.” They would predict that perceived duration for the RSVP sequence increases linearly with the number of reported targets for the No-target condition, One-target and Two-target conditions. However, in the present study, we found that the number of perceived targets, which Herbst et al. (2012) took as an index of attentional selection, did not determine the perceived duration or perceived frame numbers; both post hoc tests after ANOVA and additional Bayes factor analyses found no significant difference between the One-target condition and the Two-target condition with no error. Rather, we found that successes (or failures) in detection, identification, and registration of target(s) would cause the inflation (or deflation) of both perceived temporal duration and perceived frame numbers. Especially, processing to proper accomplishment of target detection (by detecting the whole targets in RSVP sequence) would inflate both the perceived temporal duration and perceived frame numbers. However, if that target detection was not properly accomplished (by missing the last target), perceived temporal duration and perceived frame numbers would be at the same level with or less than the No-target condition in which properly no target was detected.

The present results demonstrate that conducting a nontemporal task (target detection) affects the perceived temporal duration. It has been assumed that concurrent temporal task and nontemporal task would share the same limited attentional resources (e.g., Brown, 1985; Buhusi & Meck, 2009). This assumption was supported by reports that, in dual-task paradigms, perceived duration is deflated when a distracting nontemporal task takes up more attentional resources (Brown, 1997; Casini & Macar, 1997). In the present Experiment 1, participants conducted dual task in viewing RSVP sequences: duration judgment and target detection. We have to discuss whether limited attentional resource in terms of the dual task affects the perceived duration in this study. In the One-target condition and Two-target condition with no error, we found inflation for perceived duration, rather than reduction, which was expected in terms of limited resource. Therefore, we cannot explain the inflation of perceived duration in these conditions in terms of limited attentional resource. In the Two-target condition with AB, we found that perceived duration was reduced compared with the duration perceived in the Two-target condition with no error. In this case, as participants failed to conduct the nontemporal task (detection of the second target), we cannot attribute the reduction of perceived duration to limited attentional resource, which is caused by nontemporal task. We have to consider factor other than limited attentional resource as a cause for inflation and reduction of the perceived duration in the present study.

As mentioned previously, we found that there was no difference between the One-target condition and the Two-target condition with no error. This result implies that not simply the number of detected targets or target search itself, but maybe cognitive load, which is imposed on the visual system to detect, identify, and register the whole targets in the RSVP sequence would inflate both the perceived duration and perceived frame numbers in viewing RSVP sequence. Previous studies have found that cognitive load, or physical efforts, which are involved in conducting nontemporal tasks would affect the perceived duration. For instance, a meta-analysis involving more than 100 studies revealed that cognitive load affects temporal duration judgments (Block et al., 2010). That is, for retrospective time judgment, the perceived duration under high cognitive load tends to be longer than under low cognitive load, while, for prospective time judgments, the perceived duration under high cognitive load tends to be judged shorter than that under low cognitive load. Lu et al. (2011) reported that heavier weights were judged to last longer than lighter ones in the kilogram range for retrospective test although this effect was lost for lighter (gram range) and physical load would be irrelevant for action (ton range) range. These previous studies, together with the present results, suggest that observers’ efforts to accomplish a task, regardless of whether cognitive or physical, would elongate the perceived temporal duration. Previous studies have found that stimuli with larger magnitudes in nontemporal dimensions, such as number of dots, size of open squares, luminance of solid squares, and numeric value of digits, would be judged to last temporally longer (Xuan et al., 2007). Based upon the results of observations, one may assume a generalized magnitude system which can summarize the common property of magnitudes in various dimensions (e.g., space, time, and quantity; Lu et al., 2011; Xuan et al., 2007). The present results suggest that, not only the magnitudes in nontemporal stimulus dimensions but also the cognitive load, which is imposed on the visual system to process a stimulus, might be involved in such a generalized magnitude system as suggested by a previous study (Lu et al., 2011). If the visual system cannot find any reliable information concerning a particular temporal duration, then it might rely on any salient information from the generalized magnitude system concerning any kind of magnitudes involved in processing stimuli to estimate the duration.

Not only cognitive load to accomplish the nontemporal task but also state of awareness of accomplishment (or failure) of the task might cause the difference in perceived duration in viewing the Two-target conditions with AB and with no error. Kanai et al. (2010) showed that participants could distinguish missing targets from the physical absence of targets in terms of AB. That is, participants’ confidence levels are higher for the correct rejections when no target was presented than for missing targets that are presented, as with the AB. In contrast, such a distinction was not found for the failure of target detection in terms of contrast reduction, backward masking, and flash suppression, in which they missed the target as a consequence of reduction in the sensory signal level. These results suggest that, if participants did not detect the second target, there are some differences in subjective states of awareness between miss of T2 (AB), in which participants failed to detect the second target for the Two-target condition, and correct rejection, in which they did not detect the second target, and they correctly deny the presence of the second target for the One-target condition. If they are aware of their own missing T2, which is delivered from an AB, or if they aware of accomplishing the target detection in viewing RSVP sequences for the One-target condition, these states of awareness between the AB and correct rejection (Kanai et al., 2010) would affect the perceived duration; awareness of accomplishing the target detection for the One-target and Two-target conditions, regardless of number of targets, may inflate the perceived duration, while awareness of missing the last target in RSVP sequence would reduce the perceived duration.

The main difference in the effects for the perceived duration (Experiment 1) and number of perceived frames (Experiment 2) was the effects of the actual frame differences between the comparison and standard sequences; the perceived frame number increased with increment of the actual frame difference, while the perceived duration is hardly affected by the actual frame difference. We have to discuss the basis for this difference between perceived duration and perceived frame number. At first, we should notice that the actual frame difference between standard and comparison sequences used in this study might be too small to detect its effects on perceived duration. However, we found effects of the actual frame difference on perceived frame number. These results of two experiments suggest that the duration perception would be more sensitive to the number of actually presented frames than frame number perception. This difference in sensitivity to actually presented frame number would be caused by relationship among those factors. That is, the perceived frame number would be directly derived from the number of actually presented frames, while the perceived duration would be indirectly derived from the number of actually presented frames, maybe through the generalized magnitude system (Lu et al., 2011; Xuan et al., 2007). Future study has to assess these assumptions by directly comparing the effects of frame number on perceived duration with those on perceived frame number.

Exogenous attention, which is introduced by transient luminance change, may delay the visual processing (Hein et al., 2006; Herbst et al., 2014; Rolke et al., 2008; Yeshurun & Levy, 2003). Therefore, one may expect that the attention introduced by each frame in an RSVP sequence would delay the perceived offset of the RSVP sequence, and this delay would cause the inflation of both the perceived duration and number of perceived frames for the One-target and Two-target conditions. However, this expectation does not fit to the present findings. That is, we found no inflation of the perceived duration or perceived number of frames in viewing the No-target condition in which each frame might attract the exogenous attention by luminance change. In fact, in viewing the RSVP sequence with the No-target condition, participants attended properly to the display to detect targets as shown by relatively false alarm rate in this condition (5.2% in Experiment 1, and 6.8% in Experiment 2) compared with the false alarm rate for the second target in the one-target condition (4.7% in Experiment 1, and 16.7% in Experiment 2). These results indicate that the inflation of the perceived duration and perceived number of frames would be caused by factors, which were related to processing of target detection (such as cognitive load imposed on the visual system) rather than by exogenous attention to the RSVP sequence. In addition, the attention involved in target detection, rather than that introduced by onset of distractor, might contribute to the inflation of the perceived duration for the One-target and Two-target conditions in which the whole targets were successfully detected. That is, target detection would open an attentional gate (Reeves & Sperling, 1986), while the AB does not allow for an attentional gate to be opened. This opening the attentional gate would cause the inflation of the perceived duration for the One-target and Two-target conditions without error.

Although understanding of the basis of the AB is not the main purpose of this study, we should discuss the compatibility of the present results with several models for the AB. Unfortunately, no model so far proposed for the AB can explain the whole results reported here. On one hand, the models which depend upon capacity limitation (e.g., Chun & Potter, 1995; Shapiro et al., 1994) have difficulty in explaining small difference in perceived duration between the No-target condition, which should undergo no capacity limitation to detect targets, and the Two-target condition with AB, which should undergo capacity limitation in processing targets. On the other hand, the models that are free from capacity limitations (e.g., Olivers & Meeter, 2008; Wyble et al., 2009, 2011) have difficulty in explaining the similar results for the One-target condition and the Two-target condition with no error because these models assume distinct processing for each detected target, and therefore, they expect that the perceived duration increases with the number of detected targets. In addition, these models have difficulty in explaining the large difference between the Two-target condition with AB and the One-target condition because the number of detected targets, as well as number of inhibitions after the target detection, was the same in these conditions. In the present study, we found that detection of the whole targets in a RSVP sequence would inflate both the perceived duration and perceived number of frames. Based on these findings, the model for the perception of the temporal duration in viewing RSVP sequence should rely upon the accomplishment of target detection rather than the number of detected targets. Of course, these properties are not necessary for the models to explain the basis of the AB. However, future synthetic models for the visual processing in viewing RSVP sequences whose scope includes not only the target detection but also other perceptual aspects, such as perception of temporal duration and perception of event frequency, should consider these properties.

## Conclusions

We investigated the effects of detecting targets and missing them while viewing RSVP sequences upon perceived temporal duration and perceived frame number for these sequences. We found that both perceived duration and frame numbers were overestimated when targets are correctly detected regardless of the actually presented target numbers. If the second target was missed (AB occurs), the perceived duration is almost the same length with the condition in which no target was presented. Perceived temporal duration was hardly affected by actually presented frame numbers although the perceived frame number was affected by it. The cognitive loads imposed on the visual system to process properly the whole targets in RSVP sequence would serve as a determinant for perceived temporal duration in viewing the sequences.
